# Targeting Super-Enhancers as a Therapeutic Strategy for Cancer Treatment

**DOI:** 10.3389/fphar.2019.00361

**Published:** 2019-04-11

**Authors:** Yi He, Wenyong Long, Qing Liu

**Affiliations:** Department of Neurosurgery, Xiangya Hospital, Central South University, Changsha, China

**Keywords:** super-enhancer, neoplasms, bromodomain and extra-terminal domain protein, cyclin-dependent kinase 7, enhancer elements

## Abstract

Super-enhancers (SEs) refer to large clusters of enhancers that drive gene expressions. Recent data has provided novel insights in elucidating the roles of SEs in many diseases, including cancer. Many mechanisms involved in tumorigenesis and progression, ranging from internal gene mutation and rearrangement to external damage and inducement, have been demonstrated to be highly associated with SEs. Moreover, translocation, formation, deletion, or duplication of SEs themselves could lead to tumor development. It has been reported that various oncogenic molecules and pathways are tightly regulated by SEs. Moreover, several clinical trials on novel SEs blockers, such as BET inhibitor and CDK7i, have indicated the potential roles of SEs in cancer therapy. In this review, we highlighted the underlying mechanism of action of SEs in cancer development and the corresponding novel potential therapeutic strategies. It is speculated that targeting SEs could complement the traditional approaches and lead to more effective treatment for cancer patients.

## Introduction

The hallmarks of cancer, such as aberrant proliferation, invasion, metastasis, and apoptotic evasion, are closely related to aberrant gene expression (Hanahan and Weinberg, 2011). Therefore, genetic and epigenetic changes are fundamental mechanisms of cancer (Bradner et al., 2017). Promoters refer to sites to which the basal transcription machinery is recruited, usually located within 100–1,000 bp upstream of the transcription start sites (TSS). Since a promoter usually induces basal or limited levels of gene expression, higher levels of gene expression require highly regulated promoter–enhancer interactions (Carter et al., 2002).

Enhancers refer to transcription factors (TFs) that bind to DNA regulatory elements. They play key roles in the regulation of cell-type-specific gene expression, over both short and long distances, independent of their position and orientation with respect to TSS (Banerji et al., 1981; Bulger and Groudine, 2011). They exhibit three main characteristics. First, they often contain conserved DNA sequences and are located in open chromatin regions without nucleosomes, which allows for binding of RNA polymerase, TFs, and co-activators. Second, enhancers are typically enriched with a post-translational modification histone mark, such as acetylation at H3 lysine 27 (H3K27ac) and mono-methylation at H3 lysine 4 (H3K4me1). Third, unlike promoter sequences, enhancers can be located distantly from the TSS of their target genes (from less than 10 kb to more than 1 Mb) (Hnisz et al., 2013).

Super-enhancers (SEs) comprise of a set of enhancers spanning across a long range of genomic DNA, with some individual constituent enhancers exhibiting stronger transcriptional activation ability than others (Hnisz et al., 2013; Shin, 2018). SEs exhibit a similar mechanism of action as normal enhancers. Binding of TFs to enhancers facilitates enhancer interaction with the basal transcription machinery, RNA polymerase II, and promoters in a gene-specific manner, which is mediated by “looping” of the loaded enhancer to the cognate promoter. Then, the basal transcription machinery is recruited to promoters, which initiates downstream transcription (Sengupta and George, 2017).

**FIGURE 1:**
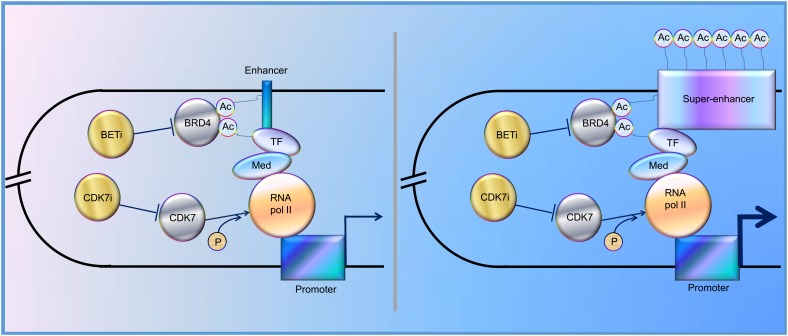
Schematic representation of the functions of enhancer and super-enhancer (SE) in the regulation of gene expression, mediated by “looping.” BRD4 binds to acetylated lysines (Ac) in enhancer, SE, and transcription factors (TF), bringing them together and mediating transcriptional co-activation and elongation via RNA polymerase II (RNA pol II) and mediator (Med) (Sengupta and George, 2017; Donati et al., 2018; Hajmirza et al., 2018). CDK7 can activate RNA Pol II by promoting its phosphorylation (Nilson et al., 2015). CDK7i, CDK7 inhibitor; BETi, BET inhibitor; p, phosphate group.

In the past decade, increasing evidence has revealed that SEs play a vital role in tumorigenesis, indicating that SEs could be one of the promising therapeutic targets for cancer treatment. Indeed, BRD4, one of the bromodomain and extra-terminal domain (BET) protein family members, binds acetylated histones at TFs, TSS, and SEs, brings them together, and mediates transcriptional co-activation and elongation via RNA polymerase II and a mediator (Hajmirza et al., 2018). Their inhibition disrupts the communication between SEs and their target promoters along with a subsequent cell-specific-repression of oncogenes, which is considered to be the main mechanism of sensitivity to BET inhibitor (BETi) (Donati et al., 2018). Besides, CDK7 inhibitor, another kind of SE blocker, functions by inhibiting phosphorylation of RNA polymerase II (Nilson et al., 2015), and has been proved to significantly inhibit tumor growth (Figure 1). Here, we review the regulation and roles of SEs in various cancers to elucidate possible therapeutic targets for cancer treatment and provide potential future directions for the studies on SEs.

## SEs in Hematological Malignancy

Table 1 summarizes the regulation and roles of SEs in hematological malignancies.

**Table 1:** SEs’ roles in hematological malignancy.

Disease^†^	Phenotype^‡^	Upstream (O/S) and potential therapeutic targets^§^	Regulation of SEs	Downstream	References
AL	DLT	BETi: OTX015 (S)	↓	NM	Berthon et al., 2016
AML	P, A	NPM1; BETi: I-BET151 (S)	↓	NM	Dawson et al., 2014
AML	D, P, G	CDK8, 19 (O)	↓	STAT1 S727	Nitulescu et al., 2017
AML	D	NCD38 (S), LSD1 (O)	↓/↑	GFI1, ERG	Sugino et al., 2017
AML	A	BETi: BI894999 (S)	↓	p-Ser2 RNA polymerase II	Gerlach et al., 2018
B-ALL	D	IKAROS (S)	↓	Sykb, CD79b	Hu Y. et al., 2017
B-ALL	A, P, D, T	STAT5 (O); PAX5, EBF, IKAROS (S)	↑/↓	NM	Katerndahl et al., 2017
BL and IL	A	EBV (O)	↑	CFLAR, IRF2	Ma et al., 2017
BPDCN	A	TCF4 (O), BETi: JQ1 (S)	↓	NM	Ceribelli et al., 2016
CLL	A, TG	SNP rs539846 (O)	↓	BCL2-BMF	Kandaswamy et al., 2016
CLL	P	PAX5 (O)	↑	BCL2, CXCR4, CD83…	Ott et al., 2018
DLBCL and FL	D	CREBBP (S)	↓	BCL6, MEF2B, MEF2C…	Zhang et al., 2017
ETP-ALL	D	NPi:GSIs (S)	↓	MYC	Knoechel et al., 2015
LP	DLT	BETi: OTX015 (S)	↓	NM	Amorim et al., 2016
LP and LK	P	CREBBP/EP300 (O); CBP30 (S)	↑/↓	GATA1, MYC	Garcia-Carpizo et al., 2018
MM	P	JQ1	↓	MYC	Loven et al., 2013
MM	DLT	BETi: OTX015 (S)	↓	NM	Amorim et al., 2016
PEL	P, A	IMiDs, JQ-1, IBET151, PFI-1 (S)	synergy	IRF4, IKZF1 (but not IKZF3)	Gopalakrishnan et al., 2016
T-ALL	TG	NOTCH1 (0)	↑	MYC	Herranz et al., 2014
T-ALL	P, A	THZ1 (S)	↓	RUNX1	Kwiatkowski et al., 2014
T-ALL	D, TG	TAL1/SCL (O)	↓	GIMAP	Liau et al., 2017

*^†^Abbreviation for cancers: AL, acute leukemia; AML, acute myelocytic leukemia; B-ALL, B-cell acute lymphoblastic leukemia; BL, Burkitt lymphoma; BPDCN, blastic plasmacytoid dendritic cell neoplasm; CLL, chronic lymphocytic leukemia; DLBCL, diffuse large B cell lymphoma; ETP-ALL, early T-cell progenitor acute lymphoblastic leukemia; FL, follicular lymphoma; IL, immunosuppression-related lymphomas; LK, leukemia; LP, lymphoma; MM, multiple myeloma; PEL, primary effusion lymphoma; T-ALL, T-cell acute lymphoblastic leukemia. ^‡^Abbreviation for phenotypes: A, apoptosis; D, differentiation; DLT, dose-limiting toxicity; G, growth; MT, metastasis. NM: not mentioned; P: proliferation; T: transformation; TG: tumorigenesis; ^§^Abbreviation for molecules: BETi, bromodomain inhibitor; EBV, Epstein-Barr virus; IMiDs, immunomodulatory drugs; NM, not mentioned; NPi, Notch pathway inhibitor; O, oncogenic; S, suppressor of cancer.*

Several mechanisms of tumorigenesis in hematopoietic system have been proved to be associated with SEs, including mutation, fusion and expression of specific genes, activation of pathways, and infection of Epstein-Barr virus (EBV).

Several mechanisms of tumorigenesis in hematopoietic system have been proved to be associated with SEs, including mutation, fusion, and expression of specific genes, activation of pathways, and infection of EBV (Herranz et al., 2014; Kandaswamy et al., 2016; Hu Y. et al., 2017; Katerndahl et al., 2017; Liau et al., 2017; Ma et al., 2017; Nitulescu et al., 2017). Generally, SEs were thought to promote tumorigenesis and malignancy, based on the reports that SEs upregulate oncogenes whereas broad H3K4me3 peaked at tumor suppressor genes (Hnisz et al., 2013; Chen et al., 2015). However, Cao et al. (2017) showed some overlaps in these two types of elements, suggesting that SEs could play both roles. Therefore, researchers should pay attention to the possible downregulation of suppressor genes when using BETi.

BET inhibitor is a hot topic in SE research. BETi, such as I-BET151 and JQ1, downregulated SE-associated genes, and suppressed proliferation and promoted apoptosis in AML multiple myeloma, acute leukemia, lymphoma, and primary effusion lymphoma (Loven et al., 2013; Dawson et al., 2014; Pelish et al., 2015; Gopalakrishnan et al., 2016). Besides, after determining dose-limiting toxicity of OTX015 in phase 1 clinical study, researchers reported that for further phase 2 studies, the once-daily recommended dose for oral, single agent use of OTX015 in patients with acute leukemia or lymphoma is 80 mg, on a 14 days on/7 days off schedule (Amorim et al., 2016; Berthon et al., 2016). Researchers also reported some new BETi, such as BI894999, and other SE inhibitors, including THZ1, NCD38, PLX51107, GSIs, and CBP30 (Kwiatkowski et al., 2014; Knoechel et al., 2015; Sugino et al., 2017; Garcia-Carpizo et al., 2018; Gerlach et al., 2018; Ott et al., 2018). These could provide novel alternatives or synergetic BETi drugs for cancer treatment. Besides, researchers have found some feedback regulations between SEs and corresponding genes. For example, STAT5 and PEPII promote the expression of corresponding SEs in B-cell acute lymphoblastic leukemia (Katerndahl et al., 2017), which indicated a better response to SE blockers in this cancer. Intriguingly, Zhang et al. (2017), Garcia-Carpizo et al. (2018) drew contradictory conclusions with each other about whether CREBBP, an acetyltransferase, promotes or suppresses cancer development. However, their conclusion came from different tumor models (diffuse large B cell lymphoma and follicular lymphoma for Zhang; leukemia and lymphoma for Garcia) and involved different downstream genes (BCL6, MEF2B, and MEF2C for Zhang; GATA1 and MYC for Garcia). Thus, the inconsistent results might be obtained due to the diverse functions of downstream genes controlled by corresponding SEs.

## SEs in Nervous System Neoplasms

Table 2 summarizes the regulation and roles of SEs in nervous system neoplasms.

**Table 2:** SEs’ roles in neoplasms of nervous system.

Cancer type^†^	Phenotype^‡^	Upstream (O/S) and potential therapeutic targets^§^	Regulation of SEs	Downstream	References
GBM	NM	NM	translocated in	TERT	Francis et al., 2014
GBM	P	NM	enriched with 5hmC	proliferation-associated TFs	Johnson et al., 2016
GBM	P	CDK7i: THZ1 (S)	↓	WNT7B, FOSL1, FOXL1…	Meng et al., 2018
NB	OS	TERT rearrangement (O)	↑	TERT	Valentijn et al., 2015
NB	P, D, CC	JQ1, THZ1 (S)	↓	TBX2, MYCN, FOXM1-DREAM	Decaesteker et al., 2018
NB	OS, TG	rs2168101, rs3750952 (in LMO1) (S)	↓	NM	He et al., 2018
MB	OS	Somatic variants (O)	↑	GFI1, GFI1B	Northcott et al., 2014
MB	P, TG	MLL4 (S)	↓	Dnmt3a and Bcl6	Dhar et al., 2018
EO	P, CC	JQ1, AZD1775, AZD4547 (S)	↓	PAX6, SKI, CCND1…	Mack et al., 2018

*^†^Abbreviation for cancers: EO, ependymoma; GBM, glioblastomas; MB, medulloblastoma; NB, neuroblastoma. ^‡^Abbreviation for phenotypes: CC, cell cycle; D, differentiation; G, growth; NM, not mentioned; OS, overall survival; P, proliferation; TG, tumorigenesis. ^§^Abbreviation for molecules: CDK7i, CDK7 inhibitor; MLL4, H3K4 methyltransferase; NM, not mentioned; O, oncogenic molecule; S, suppressor of cancer; TERT, telomerase reverse transcriptase; TF, transcription factor.*

In nervous system tumors, SEs could be regulated by gene rearrangement, single nucleotide polymorphism (SNP), binding of TFs, or modification of enzymes (Northcott et al., 2014; Valentijn et al., 2015; Dhar et al., 2018; He et al., 2018). Besides, SEs themselves could also be modified or translocated to new loci and exhibit different activities, leading to novel mechanisms for SEs-related tumorigenesis (Francis et al., 2014; Johnson et al., 2016). Furthermore, several downstream genes and related pathways of SEs have been discovered. Decaesteker et al. (2018) identified a novel core regulatory circuitry constituent (TBX2) in high-risk neuroblastoma, which was regulated by SEs. Dhar et al. (2018) reported that some SEs suppressed medulloblastoma and provided a unique tumor-suppressive mechanism in which MLL4, a H3K4 methyltransferase, is necessary to maintain broad H3K4me3 and SEs at tumor suppressor genes.

Several researchers have reported various SEs inhibitors that control cancer development. Chipumuro et al. (2014) found that CDK7i could lead to significant tumor regression in high-risk neuroblastoma mouse model without introducing systemic toxicity, which implied striking therapeutic selectivity. Henssen et al. (2016) reported that, in preclinical MYCN-driven neuroblastoma models, concurrent MYCN repression was observed in OTX015-treated samples, which could not be abrogated by ectopic MYCN expression. In addition, OTX015 treatment significantly suppressed tumor cell proliferation and improved survival of mice. Decaesteker et al. (2018) found that JQ1 coupled with THZ1 prevented the cell growth, proliferation, and differentiation in neuroblastoma primary cultured cell through strong repressive effects on CRC gene expression and p53 pathway response. Similarly, by gene mapping and integrating data with drug interaction databases, Mack et al. (2018) identified and validated dependency of ependymoma to SEs, which was responsive to SE inhibition. Meng et al. (2018) reported that THZ1 inhibits growth and proliferation of glioblastoma cells both *in vitro* and *in vivo*. In addition, CDK7 inhibition via CRISPR-Cas9 or RNA interference significantly disrupted GBM cell growth.

These results indicated that inhibitors of SEs could be promising candidates for cancer treatment.

## SEs in Visceral Organ Tumors

Table 3 summarizes the regulation and roles of SEs in visceral organ tumors.

**Table 3:** SEs’ roles in visceral organ tumors.

Cancer type^†^	Phenotype^‡^	Upstream (O/S) and potential therapeutic targets^§^	Regulation of SEs	Downstream	References
LC	P, G	NSD2 (O)	↑	RAS	Garcia-Carpizo et al., 2016
LC	P	TF: TBX4 (S)	↑	SFRP1, ADM, THBS1…	Horie et al., 2018
PC	NM	T2E fusion gene (O)	↑	EGR	Babu and Fullwood, 2017
CRC	G	NM	translocated on	CCAT1-L	Xiang et al., 2014
CRC	P	BETi: JQ1 (S)	↓	c-MYC	Togel et al., 2016
CRC	NM	NM	5hmC modified	NM	Hu H. et al., 2017
CRC	NM	RTD (O)	formation of a 3D contact domain	IGF2	Weischenfeldt et al., 2017
CLC	P, A	BETi: JQ1 (S)	↓	MAPK signaling pathway	Nakamura et al., 2017
PDA	P, G, I, M	KDM6A (S)	↓	DeltaNp63, MYC, RUNX3…	Andricovich et al., 2018
OSCC	P, A	CDK7i: THZ1 (S)	↓	PAK4, RUNX1, DNAJB1…	Jiang Y.Y. et al., 2017
OSCC	M, G	SCC-specific hypermethylation (O)	↓	ZFP36L2	Lin et al., 2018
OSCC	P, I, M	TF: TP63 (O)	↑	lncRNA: LINC01503	Xie et al., 2018
LIHC	P, I, M, CC	ZEB1 (O)	↑	lncRNA: HCCL5	Peng et al., 2018
CVC	P	BETi: JQ1, iBET72 (S)	↓	viral oncogenes: E6 and E7	Dooley et al., 2016
OC	OS	SNP: rs6674079 (1q22) (O)	↑	MEF2D	Johnatty et al., 2015

*^†^Abbreviation for cancers: CLC, colon cancer; CRC, colorectal carcinoma; CVC, cervical cancer; LC, lung cancer; LIHC, liver hepatocellular carcinoma; OC, ovarian cancer; OSCC, oesophageal squamous cell carcinoma; PC, prostate cancers; PDA, pancreatic ductal adenocarcinoma. ^‡^Abbreviation for phenotypes: A, apoptosis; CC, cell cycle; G, growth; I, invasion; M, migration; NM, not mentioned; OS, overall survival; P, proliferation. ^§^Abbreviation for molecules: BETi, Bromodomain inhibitor; CDK7i, CDK7 inhibitor; NM, not mentioned; O, oncogenic molecule; S, suppressor of cancer; RTD, Recurrent tandem duplications; SCC, squamous cell carcinoma; TF, transcription factor*

Most SEs promote the forming and malignancy of visceral organ tumors. For example, SEs lead to the overexpression of ERG, leading to overexpression of target genes that drive development of prostate cancer (Babu and Fullwood, 2017). Moreover, SEs activate MAPK signaling pathway to inhibit apoptosis and promote proliferation of colon cancer (Nakamura et al., 2017).

However, in some cases they also suppressed cancer development. For example, TBX4, which is highly associated with SEs, was downregulated in lung cancer-associated fibroblasts (Horie et al., 2018). DNA hypermethylation suppressed some SEs in squamous cancer cells, whose downstream genes, such as SMGs, CUL3, and ZFP36L2, are important tumor-suppressors specific to the OSCC subtype (Lin et al., 2018).

Gene mutation, gene fusion, and aberrant expression of oncogenes or TFs activate SEs and ultimately lead to tumorigenesis (Johnatty et al., 2015; Garcia-Carpizo et al., 2016; Babu and Fullwood, 2017; Andricovich et al., 2018; Peng et al., 2018; Xie et al., 2018). Besides, translocation, 5hmC modification, methylation profile shifts, or 3D contact domain formation of SEs also lead to cancer development (Xiang et al., 2014; Hu H. et al., 2017; Weischenfeldt et al., 2017). The effect of SE blockers have been tested in various cancers, including the testing of THZ1 in esophageal cancer, iBET72 in cervical cancer, and JQ1 in cervical cancer, colorectal carcinoma, colon cancer, and squamous cell carcinoma (Dooley et al., 2016; Togel et al., 2016; Jiang Y.Y. et al., 2017; Nakamura et al., 2017). Thus, they could potentially be used as biomarkers or therapeutic targets in the future.

## SEs in Other Cancers

Table 4 summarizes the regulation and roles of SEs in other tumors.

**Table 4:** SEs’ roles in other cancers.

Cancer type^†^	Phenotype^‡^	Upstream (O/S) and potential therapeutic targets^§^	Regulation of SEs	Downstream	References
TNBC	A, P, G	CDK7i: THZ1 (S)	↓	EGFR, FOSL1, FOXC1…	Wang et al., 2015
TNBC	A, P	BETi: JQ1 (S)	Not involved	mitosis regulator LIN9	Sahni et al., 2017
BC	DR	AI (O)	↑	FOXO1, FOXA1, FOXA2…	Nguyen et al., 2015
BC	T, D	GER (O)	↑	KDM6A, EN1, TBX18…	Su et al., 2015
BC	IE	TNF-NFKB1 pathway (O)	↑	CD47	Betancur et al., 2017
BC	NM	RTD (O)	↑	ESR1, MYC	Glodzik et al., 2017
BC	DR	AKTi/FOXO3a/BRD4 axis (0)	↑	CDK6	Liu et al., 2018
BC	DR	apoERalpha (O)	↑	DSCAM-AS1	Miano et al., 2018
PC, PGL	NM	GR (O)	translocated on	TERT	Dwight et al., 2018
ACC	P	NM	translocated on	MYB	Drier et al., 2016
SCC	P, PG	Ets2, Elk3 (O)	↑	Ets2, Elk3, Fos, Junb, Klf5	Yang et al., 2015
SCC	NM	UVR (O)	formation	CYP24A1, GJA5, SLAMF7	Shen et al., 2017
MO	G, P	BETi: JQ1 (S)	↓	PGC-1α	Gelato et al., 2018
NPC	G	BETi: JQ1 (S)	↓	ETV6	Ke et al., 2017
NPC	P, A	CDK7i: THZ1 (S); ETS2, MAFK, TEAD1 (O)	↓/↑	BCAR1, F3, LDLR, TBC1D2G1	Yuan et al., 2017
Cancers	TG	NM	deletion	MYC	Dave et al., 2017
Cancers	P, M, I	NM	NM	linc00152	Xu et al., 2017
Cancers	NM	TADs boundaries (O)	insulated and co-duplicated	CTCF	Gong et al., 2018

*^†^Abbreviation for cancers: ACC, adenoid cystic carcinoma; BC, breast cancer; MO, melanoma; NPC, nasopharyngeal carcinoma; PC, pheochromocytomas; PGL, paragangliomas; SCC, squamous cell carcinoma; TNBC, triple-negative breast cancer. ^‡^Abbreviation for phenotypes: A, apoptosis; D, differentiation; DR, drug-resistance; G, growth; I, invasion; IE, immune escape; M, migration; NM, not mentioned; P, proliferation; PG, progression; T, transformation; TG, tumorigenesis. ^§^Abbreviation for molecules: AI, aromatase inhibitor; apoERalpha, ligands Estrogen receptor-alpha; BETi, Bromodomain inhibitor; CDK7i, CDK7 inhibitor; GER, genome epigenetic reprogramming; GR, genomic rearrangements; NM, not mentioned; O, oncogenic molecule; S, suppressor of cancer; RTD, Recurrent tandem duplications; TADs, topologically associating domains; TF, transcription factor; UVR, ultraviolet radiation.*

Super-enhancer-associated mechanisms involved in other cancers include genomic rearrangements in pheochromocytomas, genome epigenetic reprogramming and recurrent tandem duplication in breast cancer, and ultraviolet radiation and aberrant activation of proto-oncogene in squamous cell carcinoma (Su et al., 2015; Yang et al., 2015; Betancur et al., 2017; Glodzik et al., 2017; Shen et al., 2017; Dwight et al., 2018). Besides, Drier et al. (2016) found that translocation of SEs upregulated MYB in adenoid cystic carcinoma. Similarly, Dwight et al. (2018) reported that translocation of SEs promoted the expression of TERT in pheochromocytomas and paragangliomas. Besides, formation of SEs upregulated several genes, such as CYP24A1, GJA5, SLAMF7, and ETV1, in squamous cell carcinoma (Shen et al., 2017), and deletion of SEs could lead to downregulation of MYC in several tumors. (Dave et al., 2017) In addition, SEs that are flanked by strong topologically associating domains (TAD) may be exploited as a functional unit to promote gene expression, and strong TAD boundaries and SE elements are frequently co-duplicated in cancer cells (Gong et al., 2018). All these studies further deepened our understanding about the mechanism of action of SE in cancers and provided novel possible targets for anti-tumor therapy.

Several researchers have widely used SE blockers to illustrate the implication of SEs in cancers. Wang et al. (2015), Sahni et al. (2017) investigated and verified the efficacy of THZ1 and JQ1 to inhibit proliferation and promote apoptosis of cancer cells in triple-negative breast cancer model. Similarly, Ke et al. (2017), Yuan et al. (2017) used nasopharyngeal carcinoma model to explore the efficacy of JQ1 and THZ1, and found significant inhibition of proliferation and enhancement of apoptosis. JQ1 was also reported to reduce the cell proliferation in melanoma model (Gelato et al., 2018). However, it is noteworthy that sometimes BETi could still function without participation of SEs. Sahni et al. (2017) reported that in triple-negative breast cancer, the mitosis regulator, LIN9, was often amplified and overexpressed. Although, it lacked a related SE, BETi could decrease its expression and inhibit mitosis in triple-negative breast cancer. Donati et al. (2018) reported that BRD4 participates in the activation and repair of DNA damage checkpoints and telomere maintenance. Therefore, in addition to blocking the function of SE, BETi can also inhibit tumorigenesis via other mechanisms, such as hindering the repair of DNA damage. This raised a question about the reliability of SEs’ roles in cancers illustrated by BETi mechanism of action in previous studies. Did the function of BETi really arise from the inhibition of SEs, or actually they work through some other pathways? We still cannot draw a definite conclusion.

Notably, SEs are involved in the drug resistance of breast cancer cells. (Nguyen et al., 2015; Liu et al., 2018; Miano et al., 2018). Although it is still not sure whether SE blockers can reverse drug resistance, they may be good research targets to find novel drugs to treat drug-resistant tumors.

## Regulation of SEs by Tumor-Associated Viruses

Table 5 summarizes the regulation of SEs by cancer-related viruses.

**Table 5:** regulations of SEs by tumor-associated viruses.

Virus^†^	Phenotype^‡^	Upstream (O/S) and potential therapeutic targets^§^	Regulation of SEs	Downstream	References
HPV	P	BETi: JQ1, iBET72 (S)	↓	E6 and E7	Dooley et al., 2016
HPV	NM	KDM5C (S)	↓	EGFR, c-MET	Chen et al., 2018
HPV	NM	NM	SELE formation	E6/E7	Warburton et al., 2018
EBV	G	EBNA2 (O)	↑	RUNX3, RUNX1	Gunnell et al., 2016
EBV	P, G	EBV nuclear antigens (O)	↑	MCL1, IRF4, EBF, MYC	Jiang S. et al., 2017

*^†^Abbreviation for viruses: EBV, Epstein-Barr virus; HPV, human papillomavirus. ^‡^Abbreviation for phenotypes: G, growth; NM, not mentioned; P, proliferation. ^§^Abbreviation for molecules: BETi, Bromodomain inhibitor; NM, not mentioned; O, oncogenic molecule; S, suppressor of cancer; SELE, super-enhancer-like element.*

Some virus-induced SE alteration in abovementioned cancers were discussed in corresponding parts of this review. Other studies, that did not involve specific tumors, found that SEs generally promoted tumorigenesis in virus-related cancers. The carcinogenic potential of viruses could arise from aberrant activation of host genes as well as integration of viral genes, both requiring the participation of SEs (Dooley et al., 2016; Gunnell et al., 2016; Jiang S. et al., 2017; Chen et al., 2018; Warburton et al., 2018). These results implied potential application of SE blockers to treat patients with high cancer risk from virus infection.

## Future Directions

Super-enhancers refer to a class of regulatory regions with unusually strong enrichment of the binding sites for transcriptional co-activators. Although the roles of individual SEs vary with downstream genes, their overall effect in a particular tumor is relatively stable. In most of the cancer cases, SEs act as oncogenes to promote tumor growth. Therefore, SEs could be a promising therapeutic target in those cancers.

**FIGURE 2:**
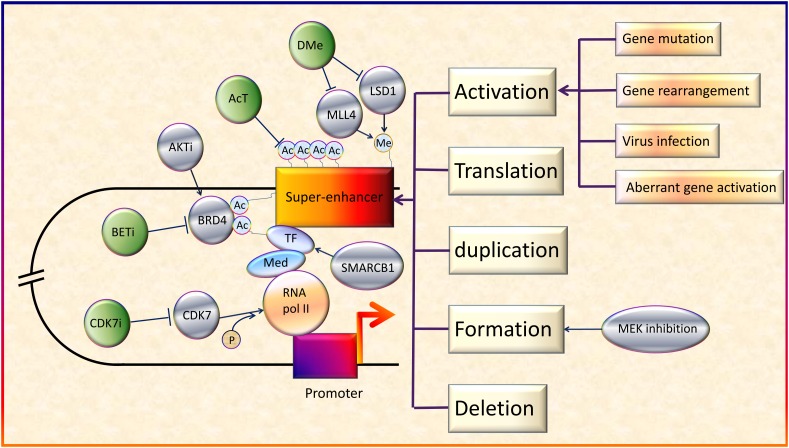
Regulation and therapeutic targets of SEs in cancers. On the whole, SEs can be activated by various genetic alterations including gene mutation, gene rearrangement, aberrant activation of genes and virus infection (Northcott et al., 2014; Kandaswamy et al., 2016; Chen et al., 2018; Dwight et al., 2018; Ott et al., 2018). In addition, activation, translation, duplication, formation, and deletion of SEs will also lead to abnormal transcription and cancer development (Xiang et al., 2014; Babu and Fullwood, 2017; Dave et al., 2017; Shen et al., 2017; Gong et al., 2018). Besides, acetyltransferase (AcT), like CREBBP/EP300, strengthens the function of BRD4 by promoting chromatin acetylation (Garcia-Carpizo et al., 2018). Demethylases (DMe), such as KDM5C, KDM6A, and lysine-specific demethylase 1 inhibitors (LSD1i), can suppress SEs via demethylation (Sugino et al., 2017; Andricovich et al., 2018; Chen et al., 2018). On the contrary, MLL4, a H3K4 methyltransferase, helps to maintain broad H3K4me3 and SEs (Dhar et al., 2018). SMARCB1, a core subunit of the SWI/SNF (BAF) chromatin-remodeling complex, helps stabilize TFs (Wang et al., 2017). MEK inhibition opens chromatin and establishes super-enhancers at genes required for late myogenic differentiation, through ERK2/MYOG pathways (Yohe et al., 2018). AKT inhibitors (AKTi) induce FOXO3a acetylation as well as BRD4 recognition (Liu et al., 2018). Although SEs generally upregulate oncogene expression, in some cases, they also promote the expression of tumor suppressor genes (Pelish et al., 2015).

Several studies to investigate the potential of SEs as therapeutic targets have been conducted. Some researchers reported the existence of positive feedback loops. For example, Ets2 and Elk3 genes in squamous cell carcinoma can upregulate specific SEs (Yang et al., 2015). This not only intensified the function of the downstream genes, but also indicated potentially more sensitive responses to SE blockers. Besides, CDK7i showed striking selectivity in regression of neuroblastoma cells, without significant systemic toxicity (Chipumuro et al., 2014). In addition, some key downstream molecules and pathways of SEs are involved in various tumors, making them promising therapeutic targets for multiple cancers (Xu et al., 2017). Importantly, the effect of BETi has been proven in some virus-induced tumors models. For example, JQ1 and iBET72 could inhibit proliferation of cervical neoplasia induced by HPV (Dooley et al., 2016). Thus, SE blockers may have the potential to treat virus-induced cancer as well as patients with high cancer risk from virus infection. According to these studies, SEs have good prospects as potential therapeutic targets for cancers because of their strong potency, high selectivity, and broad applicability (Figure 2).

However, although the roles of SEs have been validated in many cancer cells, the degree of their involvement is still controversial. The main mechanism of action of BETi is considered to be blocking of the interaction between SEs and BRD4, which is a co-activator that can bind acetylated histones in SEs and TFs and directly interact with the mediator complex and elongation factors (Dawson et al., 2011). However, some target genes of BETi, such as LIN9 gene in triple-negative breast cancer, do not possess any corresponding SEs sites (Sahni et al., 2017). In addition, some researchers have proposed a non-transcriptional role of BRD4 in activation and repair of DNA damage checkpoints and telomere maintenance, opening new perspectives on the use of BETi in cancer (Donati et al., 2018). Similarly, other than inhibiting SEs, THZ1 can also cause defects in Pol II phosphorylation, co-transcriptional capping, promoter proximal pausing, and productive elongation (Nilson et al., 2015). Therefore, the roles of SEs in the above-mentioned cancers, illustrated by BETi and CDK7i, are still unclear. Besides, it is noteworthy that targeting SEs for cancer treatment might cause significant side effects because some tumor suppressor genes will also be suppressed when blocking SEs. Therefore, more studies and better understanding of mechanisms are urgently needed before SEs could be utilized as therapeutic targets to treat specific cancers.

Therefore, for future studies of SEs, the focus could be:

(1) SEs are cell-type-specific and have the potential to be used for identification of different subtypes of cancer. Therefore, their application in precision medicine is promising. In the future studies, researchers could try to use them to distinguish subtypes of cancer and give more precise treatment strategy.
(2) For cancers that lack known genetic drivers and are recalcitrant to therapeutic development, SE sequencing and investigation may provide novel directions for significant breakthroughs.
(3) In addition to blocking BRD4, other mechanisms of BETi are worth studying to validate the previous conclusions or obtain new explanation for the reported results.
(4) The interactions between SEs and virus infection need further research. A better understanding of them might benefit not only the virus-related cancer patients, but also those who have high risk of cancer due to virus infection.
(5) Epigenetic changes on SEs can also lead to significant differences in phenotypes. Therefore, the combination of SEs and epigenetics (such as DNA methylation and acetylation) could be good research topics.
(6) Some BETi have been put into clinical trials, such as OTX015 in multiple myeloma and acute leukemia. The practical application of BETi in other tumors awaits further exploration.

## Author Contributions

YH and WL searched and wrote the manuscript. QL revised the manuscript and supervision of the entire project.

## Conflict of Interest Statement

The authors declare that the research was conducted in the absence of any commercial or financial relationships that could be construed as a potential conflict of interest.
